# Anion inhibition studies of the α-carbonic anhydrases from *Neisseria gonorrhoeae*

**DOI:** 10.1080/14756366.2021.1929202

**Published:** 2021-05-24

**Authors:** Alessio Nocentini, Chad S. Hewitt, Margaret D. Mastrolorenzo, Daniel P. Flaherty, Claudiu T. Supuran

**Affiliations:** a Dipartimento Neurofarba, Sezione di Scienze Farmaceutiche e Nutraceutiche, Università degli Studi di Firenze, Sesto Fiorentino, Italy; b Department of Medicinal Chemistry and Molecular Pharmacology, College of Pharmacy, Purdue University, West Lafayette, IN, USA; c University of California, San Diego (UCSD), San Diego, CA, USA; d Purdue Institute for Drug Discovery, West Lafayette, IN, USA; e Purdue Institute of Inflammation, Immunology and Infectious Disease, West Lafayette, IN, USA

**Keywords:** Carbonic anhydrase, inhibitor, anion, Neisseria gonorrhoeae, dithiocarbamate

## Abstract

The bacterial pathogen *Neisseria gonorrhoeae* encodes for an α-class carbonic anhydrase (CA, EC 4.2.1.1), NgCA, which was investigated for its inhibition with a series of inorganic and organic anions. Perchlorate and hexafluorophosphate did not significantly inhibit NgCA CO_2_ hydrase activity, whereas the halides, azide, bicarbonate, carbonate, stannate, perosmate, diphosphate, divanadate, perruthenate, and trifluoromethanesulfonate showed inhibition constants in the range of 1.3–9.6 mM. Anions/small molecules such as cyanate, thiocyanate, nitrite, nitrate, bisulphite, sulphate, hydrogensulfide, phenylboronic acid, phenylarsonic acid, selenate, tellurate, tetraborate, perrhenate, peroxydisulfate, selenocyanate, iminodisulfonate, and fluorosulfonate showed K_I_s in the range of 0.15–1.0 mM. The most effective inhibitors detected in this study were sulfamide, sulfamate, trithiocarbonate and *N,N*-diethyldithiocarbamate, which had K_I_s in the range of 5.1–88 µM. These last compounds incorporating the CS_2_^-^ zinc-binding group may be used as leads for developing even more effective NgCA inhibitors in addition to the aromatic/heterocyclic sulphonamides, as this enzyme was recently validated as an antibacterial drug target for obtaining novel antigonococcal agents

## 1. Introduction

The metalloenzyme carbonic anhydrase (CA, EC 4.2.1.1) is common and widespread in prokaryotes^1^^,^^2^, organisms in which it catalyses the interconversion between CO_2_ and bicabornate, thus participating to crucial physiologic processes connected with pH regulation, metabolism, acclimation of the organism in various niches in which it lives, and in the case of pathogenic organisms, also acting as a virulence factor^3^^,^^4^. Bacteria encode for four CA genetic families, the α-, β-, γ- and ι-CAs, some of which have been characterised in detail for many of the common pathogens, such as *Escherichia coli*, *Helycobacter pylori*, *Mycobaterium tuberculosis*, *Vibrio cholerae, Pseudomonas aeruginosa, Staphylococcus aureus, Porphyromonas gingivalis, Streptococcus* spp., etc.^3–10^. Considering bacterial CAs as potential drug targets for antiinfectives was already proposed some time ago^3^, and although this idea initially met resistance from the scirntific community, a range of new data show indeed that inhibiting these enzymes may lead to significant bacteriostatic or bactericidal effects, the best examples being for the moment the sulphonamide CA inhibitors (CAIs) impairing the growth of *H. pylori*^3^^,^^4^, *E. coli*^5^, *M. tuberculosis*^6^ and more recently, the vancomycin resistant enterococci (VRE)^11^. For the last pathogens, which represent a major public health threat, the sulphonamide CAIs acetazolamide and some of its derivatives, as well as dorzolamide, outperformed linezolid, the drug of choice for treating VRE infections, as shown recently by Flaherty’s group^11^. All these promising new data offer the long-awaited^12^ proof-of-concept that inhibition of bacterial CAs may lead to antibiotics with a novel mechanisms of action, less prone to the development of drug resistance, as demonstrated for *H. pylori* and ethoxzolamide, case in which a certain level of mutations were observed in several bacterial genes, including the α-CA one, but the pathogen remained susceptible to the drug at low enough, clinically relevant concentrations^4^.

*Neisseria gonorrhoeae* is a pathogenic bacterium which became a global health concern as it provokes a sexually-transmitted disease difficult to treat due to increased levels of drug resistance to a wide range of antibiotics, including last generation cephalosporins^13–15^. Recently, we have demonstrated that the α-CA present in its genome, NgCA, is a druggable target, and its inhibition with sulphonamide inhibitors based on the acetazolamide scaffold induced a significant antigonococcal activity without toxicity to the host cells^15^. As there is a stringent need for novel antigonococcal agents, a deeper investigation of this enzyme and profiling of various calsses of inhibitors may be of great interest. Here we investigate anions and other small molecules as inhibitors of NgCA, considering that these compounds represent a classical type of CAIs^16^.

## 2. Materials and methods

### 2.1. Chemistry

Anions and small molecules were commercially available reagents of the highest available purity from Sigma-Aldrich (Milan, Italy). Purity of tested compounds was higher than 99%.

### 2.2. Enzymology

NgCA was a recombinant enzyme obtained in-house as described earlier^15^.

### 2.3. Ca activity and inhibition measurements

An Applied Photophysics stopped-flow instrument has been used for assaying the CA catalysed CO_2_ hydration activity^17^. Phenol red at a concentration of 0.2 mM was used as pH indicator, working at the absorbance maximum of 557 nm, with 10 mM Hepes (pH 7.4) as buffer, and in the presence of 10 mM NaClO_4_ for maintaining constant the ionic strength, following the initial rates of the CA-catalysed CO_2_ hydration reaction for a period of 10–100 s. The CO_2_ concentrations ranged from 1.7 to 17 mM for the determination of the kinetic parameters and inhibition constants. For each inhibitor, at least six traces of the initial 5–10% of the reaction have been used for determining the initial velocity. The uncatalyzed rates were determined in the same manner and subtracted from the total observed rates. Stock solutions of inhibitors (10–20 mM) were prepared in distilled-deionized water and dilutions up to 0.01 µM were done thereafter with the assay buffer. Inhibitor and enzyme solutions were preincubated together for 15 min at room temperature prior to assay, in order to allow for the formation of the E-I complex. The inhibition constants were obtained by non-linear least-squares methods using PRISM 3 and the Cheng-Prusoff equation, whereas the kinetic parameters for the uninhibited enzymes from Lineweaver-Burk plots, as reported earlier^18–20^, and represent the mean from at least three different determinations. NgCA concentration in the assay system was of 6.5 nM.

## 3. Results and discussion

The possibility to use CAIs for inhibiting the growth of *N. gonorrhoeae* was explored *in vitro* already in the 60 s^21^, whereas only in the ‘90 s Carter’s group^22^ reported the presumed presence of CAs in these paathogens, by using a monospecific antibody prepared against the purified *Neisseria sicca* enzyme. In that study^22^ it has been shown that several all *Neisseria* spp., including various isolates of *N. sicca*, *N. gonorrhoeae*, *N. meningitidis* and *N. lactamica*, were sensitive to the sulphonamide CAI acetazolamide, but thereafter, the enzyme was purified and characterised only in 1997 by Lindskog’s group^23^, who showed that NgCA is an α-class CA possessing a high catalytic activity^23–25^, with a k_cat_ for the CO_2_ hydration reaction of 1.7 × 10^6^ s ^− 1^. In the same work it has been demonstrated that NgCA was inhibited by metal complexing anions such as cyanide, cyanate, thiocyanate, and azide (as determined by using the esterase actvity of the enzyme with 4-nitrophenyl acetate as substrate^23^), whereas the X-ray crystal structure of the enzyme alone or in complex with acetazolamide was also reported^25^. These interesting studies, which could open the era of the CAIs as antiinfectives were however not continued, probably due to the retirement of Prof. Lindskog.

**Figure 1.**
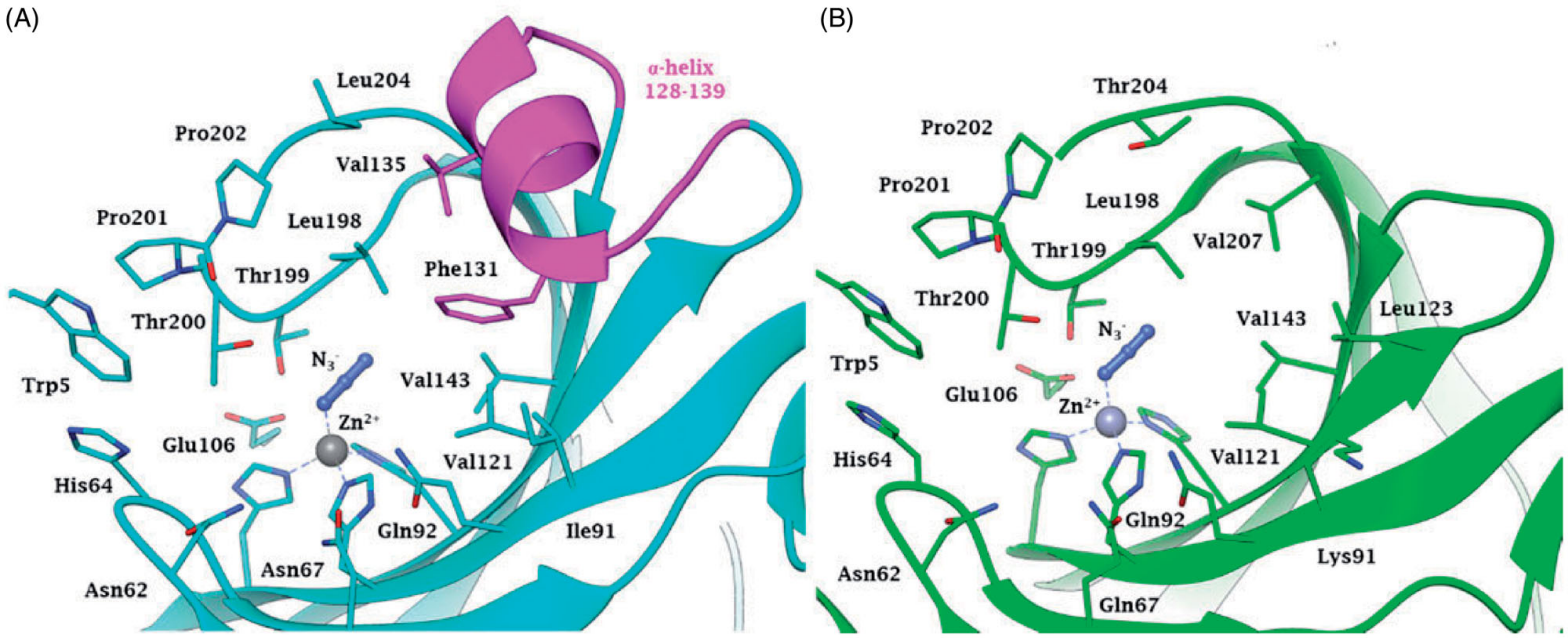
Active site view of A. hCA II (pdb 1RAY) and B. NgCA (pdb 1KOP) in adduct with the azide anion N_3_^-^. Amino acid residues of NgCA are renumbered according to the corresponding residues from hCA II. The zinc ion, represented as a grey sphere, is coordinated by three His residues, that are His94, His96 and His119, and the azide anion. Residues constituting the α-helix portion 128–139 are coloured magenta in hCA II, while being absent in NgCA.

Indeed, the tridimensional structure of NgCA complexed with the anion inhibitor azide (Figure 1) shows an active site architecture quite similar to that of the dominant human (h) isoform, hCA II. The metal ion coordination and the binding mode of the anion inhibitor are identical in the two enzymes (Figure 1(A,B)), as well as the other residues involved in catalysis/inhibition, among which the dyad Glu106 – Thr199, which orientates the substrate CO_2_ for the nucleophilic attack and participates to the binding of inhibitors too^12^^,^^25^. The main difference between the human and the bacterial enzyme is represented by the α-helix loop 128–139 (Figure 1(A)) which is present in the human and absent in the bacterial enzyme (the same situation was observed also for the α-CA from *H. pylori* and exploited to obtain inhibitors with higher affinity for the bacterial than the human enzyme^4^).

Thus, considering the scarcity of NgCA inhibition data, for which only 4 anions were investigated using the esterase activity^23^, as mentioned earlier, together with the extensive sulphonamide inhibition study reported recently by us^15^, we investigated here the inhibition profile of this enzyme with a large series of inorganic metal-complexing, simple and complex anions, as well as small molecules known to act as CAIs^12^^,^^16^, such as sulfamide, sulphamic acid, phenylboronic acid, phenylarsonic acid and *N,N*-diethyl-dithiocarbamate (Table 1). The stopped-flow, CO_2_ hydrase assay was used to measure the inhibition constants shown in Table 1, and the same data for the inhibition of the red blood cell human isoforms hCA I and II^12^^,^^16^ were also included for comparison reasons. The following should be noted regarding the NgCA inhibition profile with anions and small molecules:

**Table 1.** Inhibition constants (K_I_s) of anion inhibitors against hCA I, II and NgCA by a stopped flow CO_2_ hydration assay^17^.

	K_I_ (mM)^a^		
Anion^b^	hCA I	hCA II	NgCA
F^−^	>300	>300	8.3
Cl^−^	6	200	4.8
Br^−^	4	63	4.0
I^−^	0.3	26	9.6
NCO^−^	0.0007	0.03	0.43
NCS^−^	0.2	1.6	0.92
CN^−^	0.0005	0.02	1.0
N_3_^−^	0.0012	1.51	2.1
NO_2_^−^	8.4	63	0.59
NO_3_^−^	7	35	0.85
HCO_3_^−^	12	85	1.3
CO_3_^2-^	15	73	2.9
HSO_3_^−^	18	89	0.66
SO_4_^2-^	63	>200	0.83
HS^−^	0.0006	0.04	0.55
NH_2_SO_2_NH_2_	0.31	1.13	0.058
NH_2_SO_3_H	0.021	0.39	0.024
PhAsO_3_H_2_	31.7	49	0.74
PhB(OH)_2_	58.6	23	0.15
ClO_4_^−^	>200	>200	>100
SnO_3_^2-^	0.57	0.83	1.7
SeO_4_^2-^	118	112	0.87
TeO_4_^2-^	0.66	0.92	0.76
OsO_5_^2-^	0.92	0.95	2.3
P_2_O_7_^2-^	25.8	48	4.9
V_2_O_7_^2-^	0.54	0.57	2.8
B_4_O_7_^2-^	0.64	0.95	0.65
ReO_4_^−^	0.11	0.75	0.96
RuO_4_^−^	0.101	0.69	1.9
S_2_O_8_^2-^	0.107	0.084	0.79
SeCN^−^	0.085	0.086	0.66
NH(SO_3_)_2_^2-^	0.31	0.76	0.25
FSO_3_^−^	0.79	0.46	0.61
CS_3_^2-^	0.0087	0.0088	0.088
EtNCS_2_^−^	0.00079	0.0031	0.0051
PF_6_^−^	>100	>100	>100
CF_3_SO_3_^−^	>100	>100	5.7

^a^ Mean from 3 different assays, by a stopped flow technique (errors were in the range of ±5–10% of the reported values).

^b^ As sodium salts, except sulfamide and phenylboronic acid. Phenylarsonic acid and sulphamic acid were also used as disodium and monosodium salts, respectively.

- anions known for their lower affinity to complexate metal ions, such as perchlorate and hexafluorophosphate, did not inhibit NgCA significantly up to 100 mM concentration of inhibitor in the assay system, which is also the case for their interaction with hCA I and II, as well as many other CAs belonging to all known classes. This is also the reason why we use perchlorate at 10 mM for maintaining constant the ionic strength in the stopped-flow assays, as mentioned in Materials and methods;
- Inhibition constants in the range of 1.3–9.6 mM were measured for the following anions: all the halides, azide, bicarbonate, carbonate, stannate, perosmate, diphosphate, divanadate, perruthenate, and trifluoromethanesulfonate. The last anion is an interesting case, as it does not considerably inhibits hCA I and II (K_I_s > 100 mM) being thus an NgCA-selective (although weak) inhibitor. Among the halides, fluoride and iodide were weaker inhibitors than chloride and bromide (which was the most effective one among the halides). Bicarbonate, with a K_I_ of 1.3 mM is a rather effective inhibitor (and this substrate of the enzyme is a much weaker hCA I and especially hCA II inhibitor, see Table 1), which presumably may constitute a way to inhibit the activity of this highly effective enzyme by one of the reaction products, which might be physiologically significant. This hypothesis needs to be checked, but it is known for example that in the case of *V. cholerae*, bicarbonate, generated by the activity of the various CAs present in this pathogen, induces virulence gene expression^1^;
- the following anions showed submillimolar (cyanide had a K_I_ of 1 mM) affinity for NgCA: cyanate, thiocyanate, nitrite, nitrate, bisulphite, sulphate, hydrogensulfide, phenylboronic acid, phenylarsonic acid, selenate, tellurate, tetraborate, perrhenate, peroxydisulfate, selenocyanate, iminodisulfonate, and fluorosulfonate, for which K_I_s in the range of 0.15 – 1.0 mM were measured (Table 1). In the case of phenylboronic acid and phenylarsonic acid, there is also a selective inhibition of the bacterial enzyme versus the human isoforms, which were much less sensitive to inhibition with these two compounds. Based on these data we hypothesise that boronic acid derivatives^26^ or benzoxaboroles^27^, which were shown to act as CAIs for other enzymes, might lead to the development of more effective and possibly NgCA-selective inhibitors.
- the most effective NgCA inhibitors among the tested anions/small molecules were sulfamide, sulfamate, trithiocarbonate and *N,N*-diethyldithiocarbamate, which had K_I_s in the range of 5.1 – 88 µM. Sulfamide and sulfamate incorporate the sulfamoyl (SO_2_NH_2_) moiety found in many of the most effective aromatic/heterocyclic sulphonamides CAIs^15^^,^^16^, whereas trithiocarbonate and *N,N*-diethyldithiocarbamate incorporate another interesting zinc-binding group for the design of CAIs, the CS_2_^-^ moiety^28^, which led to the discovery of dithiocarbamates, monothiocarbamates and tritiocarbonates as effective CAIs^29^^,^^30^. The very effective inhibitory activity of these small molecules against NgCA prompt us to propose them as interesting leads for developing more effective compounds that can inhibit the activity of this enzyme and presumably interfere with the life cycle of this pathogenic bacterium.

## 4. Conclusions

NgCA, a high activity α-CA present in the genome of the bacterial pathogen N. *gonorrhoeae* was investigated for its inhibition with a series of inorganic and organic anions. Perchlorate and hexafluorophosphate, did not inhibit NgCA significantly, whereas the halides, azide, bicarbonate, carbonate, stannate, perosmate, diphosphate, divanadate, perruthenate, and trifluoromethanesulfonate showed inhibition constants in the range of 1.3–9.6 mM. Anions/small molecules such as cyanate, thiocyanate, nitrite, nitrate, bisulphite, sulphate, hydrogensulfide, phenylboronic acid, phenylarsonic acid, selenate, tellurate, tetraborate, perrhenate, peroxydisulfate, selenocyanate, iminodisulfonate, and fluorosulfonate showed K_I_s in the range of 0.15–1.0 mM. The most effective inhibitors detected in this study were sulfamide, sulfamate, trithiocarbonate and *N,N*-diethyldithiocarbamate, which had K_I_s in the range of 5.1–88 µM. These last compounds incorporating the CS_2_^-^ zinc-binding group may be used as leads for developing even more effective NgCA inhibitors, as this enzyme was recently validated as an antibacterial drug target for obtaining novel antigonococcal agents.
